# Optimization of Subsecond Estradiol Detection through Analysis of Surface–Analyte Interactions

**DOI:** 10.1021/acselectrochem.6c00007

**Published:** 2026-03-28

**Authors:** Moriah E. Weese-Myers, Vivek Subedi, Naimah El-Amin, Faith Idahosa, Manisha Fowler, Ashley E. Ross

**Affiliations:** Department of Chemistry, 2514 University of Cincinnati, 312 College Dr. 404 Crosley Tower, Cincinnati, Ohio 45221-0172, United States

**Keywords:** estradiol, carbon surfaces, fast scan cyclic voltammetry, electrode interface, neurochemistry

## Abstract

Subsecond neurochemical signaling is the fastest and one of the most diverse forms of intercellular communication employed along the neuro-immune-endocrine axis; consequently, spatiotemporally resolved approaches with high sensitivity and selectivity tailored to specific analytes are required for adequate monitoring. However, many existing electrochemical approaches are optimized solely towards catecholamine and indolamine detection, leaving a wide array of structurally diverse neuroregulators at a disadvantage with limited detection capability. Here, we use estradiol—a fast-signaling neurosteroid vital for neuroprotection that has proven difficult to detect with existing methods—as a model target analyte for optimizing direct electrochemical detection at carbon surfaces. We take a two-pronged approach to optimizing estradiol detection by examining the electrode-analyte interface from the perspective of both the carbon surface and the target molecule. We first establish the surface characteristics of diverse carbon fibers, then examine estradiol’s electrochemical behavior at each surface. Finally, we determine the contributions of estradiol’s structure to specific adsorption, elucidating subtle structural considerations to analyte-specific tailoring of carbon electrodes. By taking this multi-perspective approach, we develop a thorough understanding of the interactions between carbon sensing surfaces and estradiol for improved sensitivity and selectivity. Moreover, our approach is applicable to other neglected neurochemicals that have proven challenging to detect. This work creates a potentially broadly applicable roadmap for customizing direct monitoring at carbon surfaces through a tailored approach to optimizing the electrode-analyte interface.

## Graphical abstract

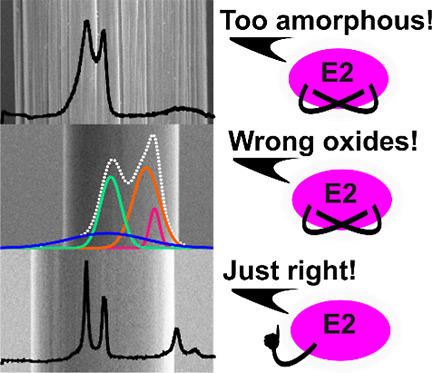

## Introduction

Estradiol (E2) is the most prolific form of estrogen and is expressed ubiquitously in the brain as a protective and regulatory factor.^1,2^ Although E2 is historically considered a circulating hormone that requires hours for genomic effects, a steadily increasing body of evidence depicts a rapid, nongenomic role in the brain. When released as a local neurosteroid, E2 effects are fast-acting through interactions at membrane receptors and directly regulate neuronal signaling.^3,4^ It induces rapid changes in dopamine signaling and modulates excitation and reward pathways.^5,6^ During and immediately after brain insult, estradiol prevents neuron death, facilitates cell proliferation, and promotes expression of anti-inflammatory factors.^7–9^ Consequently, spatiotemporally resolved monitoring of estradiol dynamics in live tissue is essential for a nuanced understanding of rapid neuroprotection. Despite significant evidence that estradiol may function on the same time scale as neurotransmission,^6,10,11^ no direct detection of subsecond E2 release has been measured in the brain due to insufficient sensing methods. This marks a significant limitation to accurate study of neuroendocrine communication.

Monitoring of neurochemical dynamics is complicated by exacting requirements for reliable sensing, requiring millisecond-to-second temporal resolution, low nanomolar limits of detection, selectivity over other rapidly-released molecules, and sufficient spatial resolution to monitor local release and minimize tissue injury. As estradiol is electroactive and easily oxidized at its primary hydroxyl, electrochemical detection methods abound. However, most existing E2 sensors focus on detection in wastewater and serum, thus prioritizing high sensitivity and selectivity while using techniques with poor temporal resolution and large sensing surfaces unsuitable for use in live tissue. In particular, electrochemical aptamer-based (EAB) sensors have successfully achieved sub-picomolar, highly selective detection of E2 in sweat and serum;^12,13^ and use of techniques such as square wave voltammetry (SWV) can provide sufficient temporal resolution for detection in tissue. However, no existing E2 sensors have demonstrated in vivo capabilities or sufficient spatial resolution for localized detection and minimal tissue damage. Here, we instead reach into our analytical toolbox and choose FSCV for highly spatiotemporally resolved detection of E2 in live tissue. FSCV employs direct electrochemical detection through application of a cyclic voltammetry waveform on the order of hundreds of volts per second, enabling low nanomolar detection with 100 ms resolution. Coupled with carbon fiber microelectrodes with diameters of 7–10 μm, FSCV causes minimal tissue damage and is frequently employed in both live tissue sections and in vivo. Our lab has previously developed a modified waveform for use with FSCV that enables nanomolar codetection of dopamine and estradiol with 100 ms temporal resolution that has been validated ex vivo in rat brain.^14^ Mariez and Trouillon demonstrated simultaneous detection of E2 and DA optimizing parameters, uncovering redox interactions and highlighting their neuromodulatory interplay.^15^ However, its decreased sensitivity to dopamine and insufficient selectivity for estradiol hinder practical use in tissue. Having reached the limits of traditional FSCV optimization, we turn from the detection technique to the electrode material to further our E2 sensing capabilities. Here, we develop a roadmap to improving detection of challenging analytes through thorough investigation of carbon surface properties, analyte-specific electrochemical interactions, and analyte structure. We take a systematic approach to subsecond E2 detection from the perspective of the electrode-analyte interface to improve rapid sensing at carbon surfaces.

Direct electrochemical detection of E2 on carbon electrodes has been used for selective and sensitive monitoring in wastewater and serum. Estradiol adsorbs strongly to graphitic carbon surfaces through a combination of π–π stacking and hydrogen bonding at oxide defect sites. Its primary oxidation is an irreversible two-step, two-electron transfer that oxidizes its phenol to a phenoxy radical to a ketone, which has sluggish electron transfer kinetics due to the ring rearrangement necessary for the second electron loss.^16,17^ At fast scan rates, electron transfer is sufficiently outstripped to resolve both single electron losses.^18^ Consequently, carbon-based E2 sensors have frequently employed nanomaterials with high conductivity and catalytic behavior to improve sensitivity and peak resolution. Recent electrodes have utilized multi-walled carbon nanotubes (MWCNTs),^19^ graphene oxide and reduced graphene oxide (GO/rGO) nanoparticles,^20,21^ polyaniline/carbon dot composites,^22^ and wrinkled mesoporous carbon nanoparticles^23^ deposited on carbon bases with poorer electrochemical characteristics for picomolar detection of E2 in aqueous media. With these considerations, it becomes evident that rapid, sensitive detection of estradiol in a tissue matrix at carbon surfaces will require an electrode interface that facilitates rapid adsorption and significantly improves electron transfer rates.

Fast-scan cyclic voltammetry is an attractive option for real-time detection of estradiol but requires significant optimization for facile detection in complex matrices. It is regularly used for subsecond detection of electroactive neurochemicals in the brain and immune organs.^24–26^ As the name suggests, FSCV scans at hundreds of volts per second, which provides millisecond temporal resolution but minimizes diffusion time during the waveform application. As FSCV analytes are almost always mass transfer limited by adsorption,^25,27^ a preaccumulation period at a held potential facilitates analyte preconcentration and enables detection of low nanomolar concentrations. The held potential determines the redox state of functional groups on the electrode surface that serve as binding sites for specific adsorption and can drive ionic targets to the electrode via an electrostatic gradient.^27,28^ However, E2 is charge neutral at physiological pH, limiting the effect of waveform optimization on surface coverage. FSCV traditionally employs amorphous carbon fiber microelectrodes, which are rich in surface oxide functionalization but lack the electrocatalytic properties common in graphitic materials. Given E2’s sluggish electron transfer rates and π–π stacking-based adsorption mechanism, this suggests that unmodified carbon fibers may be less than optimal for dynamic monitoring.

Here, we propose a generalizable approach to optimizing subsecond analyte detection through the electrode-analyte interface and apply it to E2. As described above, estradiol presents several challenges for direct detection on bare amorphous carbon fibers; chief among these are charge neutrality, sluggish electron transfer, and functional group-dependent adsorption limitation. We approach optimization through a two-pronged attack by first cataloguing surface structure and functionalization across a range of amorphous to graphitic carbon fibers. We then determine E2’s adsorption strength, speed, and surface coverage on these surfaces and analyze the impact of estradiol’s structure on adsorption and sensitivity. We utilize inexpensive and easily accessible carbon fibers, bypassing the complex synthesis process required by many E2-specific electrodes while still determining optimal surface characteristics for future development of carbon materials. By approaching estradiol optimization from the perspectives of both the electrode and the analyte, we create a clear picture of E2’s interfacial needs for fast and sensitive detection.

## Materials and Methods

### Safety Statement

No unexpected or unusually high safety risks were encountered.

### Materials and Chemicals

Unless specified, all reagents were purchased from Fisher Scientific (Fair Lawn NJ, USA). 10 mM stock solutions of 17β estradiol (E2), estrone (E1), estriol (E3), progesterone (P4), and corticosterone (CORT) (Sigma-Aldrich, St. Louis MO, USA) were made in 70% ethanol, stored at 4 °C, and used within 30 days. Daily working solutions were made by diluting stock solutions in phosphate-buffered saline (PBS) at pH 7.40 ± 0.01. PBS was made from 2 mM NaH_2_PO_4_, 10 mM Na_2_HPO_4_, 140 mM NaCl, and 3 mM KCl. All solutions were made using deionized water (Milli-Q, Millipore, Billerica MA, USA).

### Carbon Fiber Microelectrode Fabrication

Carbon fiber microelectrodes (CFMEs) were fabricated using 7 μm TS30 (also known as T-650), 6 μm MS40, and 5 μm HS40 carbon fibers (Mitsubishi Chemical Carbon Fiber and Composites Inc., Sacramento CA, USA). Briefly, carbon fibers were aspirated via vacuum into a 1.2 × 0.68 mm glass capillary (A&M Systems, Sequim WA, USA). The capillaries were then pulled using a vertical Narishige PE-22 Electrode Puller (Tokyo, Japan), forming a glass seal. The protruding fiber was trimmed to 50–100 μm under a microscope using a scalpel. Electrodes were soaked in isopropyl alcohol for 10 min before use and were backfilled with 1 M KCl.

### Material Characterization

Scanning electron microscopy (SEM) images were taken using a FEI XL30 SEM. Images were collected using an accelerating voltage of 5.00 kV, 5–6 mm away. Raman maps were collected from the sides of carbon fibers with a InVia Raman microscope (Renishaw, Gloucestershire, UK) excited by a 633 nm Ar-ion laser at 50% power with an integration time of 10 s. Spectra were collected in 0.2 μm steps in a 2 × 2 μm square. Peak fitting was performed for the D peak at 1350 cm^–1^ and the G peak at 1580 cm^–1^. For MS40 and HS40 fibers, the D/G ratio was calculated using by dividing the peak intensity of the D (disorder) peak by the intensity of the G (graphitic) peak. For TS30 fibers, the D and G peaks were convoluted, so the D/G ratio was determined using the area under the curve of the respective peaks. X-ray photoelectron spectroscopy (XPS) spectra were obtained using a Thermo Scientific Nexsa X-ray photoelectron spectrometer with a hemispherical analyzer and monochromatic Al Kα source (Wayne State University, Detroit MI, USA). Fiber samples were mounted using conductive Cu tape. Data was collected at a base pressure of 1.7 × 10^–7^ mbar with a flood gun employed for surface charge neutralization.

### Fast Scan Cyclic Voltammetry

Fast scan cyclic voltammograms (CVs) were collected using a WaveNeuro potentiostat with a 1 megaohm head stage (Pine Instruments, Durham NC, USA) and HDCV software (UNC-Chapel Hill, Mark Wightman) and a PC1e-6363 computer interface board (National Instruments, Austin TX, USA). Unless otherwise specified, the electrochemical waveform utilized scanned from −0.4 to 1.45 V and back at a scan rate of 400 V/s and a frequency of 10 Hz. For scan rates faster than 400 V/s, a 5 kHz low pass filter was used. Electrodes were allowed to equilibrate at the applied waveform for 10 min prior to measurement. Electrochemical measurements were made against a Ag/AgCl reference electrode in a two-electrode system. Analyte was delivered to the electrode via a home-built flow injection analysis system using a Fusion 200 Two-Channel Syringe pump (Chemyx, Stafford TX, USA) at a flow rate of 1 mL/min. Background subtraction was performed on all data to remove non-Faradaic and background currents by averaging 10 CVs collected from approximately 2 s before the analyte was introduced. Each data point is reported as the average current across three injections. Except where specified, all experiments were conducted using a 5 μM solution of E2.

### Statistical Analysis

All statistics were performed in GraphPad Prism 10 (GraphPad Software Inc., La Jolla CA, USA). Analysis of Raman spectra and XPS peaks was performed in OriginPro 2022 (OriginLab, Northampton MA, USA). Statistical p-values were considered significant at 95% confidence level (*p* < 0.05). Unless noted otherwise, values are reported as the mean ± standard error of the mean (SEM). For surface characterization data, *n* represents the number of fibers tested; for electrochemical data, *n* represents the number of electrodes.

## Results & Discussion

### Selection of Carbon Fibers

Polyacrylonitrile carbon fiber microelectrodes (CFMEs) used by FSCV are composed of amorphous carbon with highly heterogeneous surface functionalization. Previous work has demonstrated that the carbon fiber precursor and fabrication method impact adsorption kinetics and surface affinity for catecholamine and indolamine detection.^29^ Post-fabrication treatment to adjust tensile strength of carbon fiber, thereby increasing their Young’s modulus, alters the surface structure which we proposed would directly impact analyte adsorption. The tensile strength of the fibers is incidental to our goal: accessible and easily-comparable carbon fibers with differing surface characteristics that impact electrochemical detection. High Young’s modulus carbon fibers have demonstrated similar antifouling properties to MWCNT electrodes attributable to changes in surface oxide functionality.^30,31^ Here, we exploit these changes to study E2 interactions at carbon surfaces ranging from amorphous to graphitic. We utilize three fibers of differing Young’s moduli: TS30 fibers, which are highly amorphous, have low tensile strength (34 msi), and are traditionally used for FSCV; MS40, which have higher tensile strength (49 msi), and HS40, which have the highest Young’s modulus (62 msi) and exhibit graphene-like improvements in electron transfer kinetics.^30^ All carbon fibers were produced by a single company using similar manufacturing processes, are readily accessible, and inexpensive and easy to work with compared to in-house tailored fiber synthesis. By employing carbon fibers, FSCV’s traditional detection substrate, with varying physical and chemical properties, we can explore the surface characteristics necessary for optimized estradiol detection for development of analyte-specific carbon materials.^32^

### Surface Characterization

We first approach the electrode-analyte interface by determining the surface properties of each carbon fiber. Surface morphology was qualitatively assessed using scanning electron microscopy (SEM) to determine topological features on the fiber surface (Figure 1A–C). TS30 fibers are known to be amorphous^30^ and show significant variation across their surface. Extensive striation creates longitudinal peaks and valleys that increase the electrochemical surface area available for analyte adsorption and oxidation. TS30 fibers are also the largest of the three materials assessed with an average diameter of 8.5 μm. MS40 fibers were much smoother than TS30 fibers but still showed visible striations. They are also thinner, averaging 6 μm in diameter. Likewise, the HS40 fibers were thin at a 5 μm diameter and showed minimal striation, providing a smooth, uniform electrode surface. The smaller diameter and decreased surface roughness observed on the MS40 and HS40 fibers contribute to minimizing electrode surface area; therefore, differences in sensitivity to E2 across the three fibers cannot be solely attributed to changes in surface morphology and functionalization. Consequently, we normalize sensitivity to each fiber’s average geometric surface area during electrochemical investigation.

**Figure 1:**
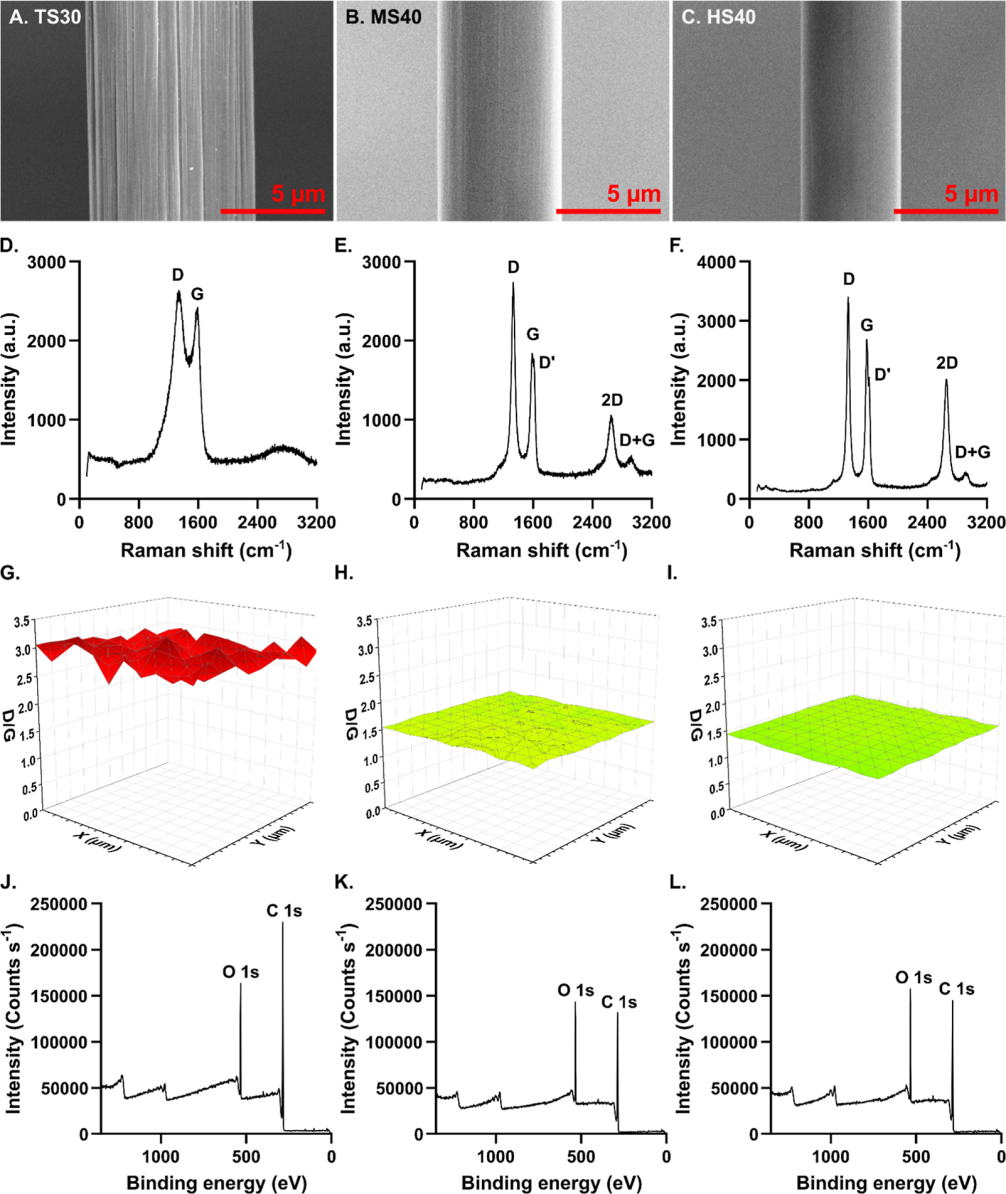
TS30, MS40, and HS40 fibers were characterized via SEM, Raman spectroscopy, and XPS. (A–C) TS30 fibers had the largest diameter and were striated, while MS40 and HS40 fibers were thinner and smooth. (D–F) Raman spectra were analyzed for D and G peaks. TS30’s peaks were convoluted, while MS40 and HS40’s peaks were clearly separable. D′, 2D, and D + G peaks also appeared for the MS40 and HS40 spectra. (G–I) Mapping the D/G ratio across each fiber revealed significant structural disorder on TS30 fibers while MS40 and HS40 fibers were highly structured. (J–L) XPS analysis showed changes in oxide functionalization between each fiber without changing overall oxygen content.

Carbon disorder and surface uniformity were investigated using Raman spectroscopy. TS30 fibers are highly heterogeneous and are traditionally considered amorphous carbon. Previous work in our lab has demonstrated that increased Young’s modulus corresponds to higher graphitic character on carbon fiber surfaces, with HS40 fibers demonstrating significant crystallinity compared to the amorphous TS30.^30^ The TS30 spectrum shows high convolution of the D peak (1350 cm^–1^) and G peak (1580 cm^–1^) with no apparent secondary peaks (Figure 1D). The absence of sharp, clearly separated bands indicates low crystallinity and resembles spectra for tetrahedral α-carbon structures.^33,34^ Conversely, D and G peaks on the HS40 spectrum are almost completely deconvoluted (Figure 1F). Several secondary peaks emerge on the HS40 spectrum, most significantly a 2D peak at 2700 cm^–1^, which indicates that the highly ordered, graphitic structure continues beyond a single layer on the fiber surface.^35^ The MS40 D and G bands are mostly deconvoluted but are broader than those observed on the HS40 spectrum, and while a 2D peak is present, it is significantly smaller than the HS40’s (Figure 1E). A D + G peak was also observed at 2950 cm^–1^, consistent with its appearance in graphene.^36^ To determine the proportion and uniformity of disordered to structured carbon across the fiber surface, fibers were mapped in a 2 μm × 2 μm area with 0.2 μm steps (Figure 1G–I). The D and G peaks were deconvoluted and fitted, then used to obtain the D/G ratio. TS30 fibers had the highest D/G ratio on average at 2.295 ± 0.092, indicating a high degree of structural disorder (Figure S1). Comparatively, the MS40 fiber ratio was much smaller at 1.463 ± 0.021, which was not significantly different from the HS40 ratio at 1.320 ± 0.018 (Figure S1, one-way two-tailed ANOVA with Bonferroni post hoc, *p* < 0.0001 all comparisons, *n* = 6 all fibers). D/G ratios were calculated from the band intensity for MS40 and HS40 fibers and from band area for the TS30 as appropriate for disordered carbon.^33,34^ Analysis by area artificially inflated the MS40 and HS40 D/G ratios, falsely implying higher disorder, while the TS30 ratio was halved (Figure S2). We hypothesize that MS40 and HS40 fibers will be more conducive to rapid electron transfer kinetics compared to TS30 electrodes, given their highly graphitic character.

Surface functionalization was investigated using X-ray photoelectron spectroscopy (XPS) to determine the oxide moieties present on each fiber’s surface. The C 1s (284–289 eV) and O 1s (529–534 eV) peaks were analyzed to establish the degree of oxygen content on each fiber type (Figure 1J–L). The survey spectra showed no evidence of a strong nitrogen peak for any of the fibers, indicating surface groups are exclusively carbon- and oxygen-based. Oxygen content was determined from the integrated area of the C 1s and O 1s peaks (Table 1). Total oxygen content did not vary significantly between the TS30, MS40, and HS40 fibers (Figure S3, one-way two-tailed ANOVA with Bonferroni post hoc, *p* = 0.1154 *n* = 7 all fibers). TS30 fibers contained the least oxygen at 17.7 ± 0.8%, while MS40 fibers and HS40 fibers averaged slightly higher at 19.6 ± 0.8% and 20.5 ± 1.0% respectively. TS30 fibers are traditionally considered oxide-rich and their oxygen-containing defect sites are necessary for many FSCV analytes to adsorb.^27,37–39^ Previous work at MS40 and HS40 fibers did not examine oxygen content but hypothesized that lower oxide functionalization may contribute to the anti-fouling properties observed on these fibers, similar to the effects seen on pristine CNTs.^30,31^ The high oxygen content observed here suggests that MS40 and HS40 fibers likely contain defect sites equivalent to TS30 fibers and may more accurately be considered analogous to graphene oxide rather than pure graphene.

**Table 1:**
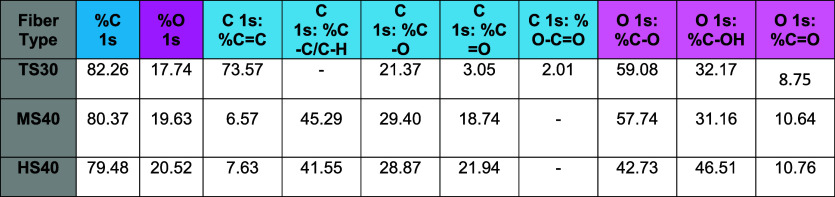
Carbon and Oxygen Functional Groups from XPS^a^

fiber type	% C 1s	% O 1s	C 1s: % C=C	C 1s:% C–C/C–H	C 1s:%C–O	C 1s:% C=O	C 1s:% O–C=O	O 1s: % C–O	O 1s: % C–OH	O 1s: % C=O
TS30	82.26	17.74	73.57	-	21.37	3.05	2.01	59.08	32.17	8.75
MS40	80.37	19.63	6.57	45.29	29.40	18.74	-	57.74	31.16	10.64
HS40	79.48	20.52	7.63	41.55	28.87	21.94	-	42.73	46.51	10.76

a Notably, TS30 fibers do not possess a C-C/C-H peak, which is the strongest C 1s peak for both MS40 and HS40 fibers.

Deconvolution of the C 1s and O 1s peaks elucidates subtle nuances in surface moieties that may selectively contribute to E2 adsorption. Four peaks were fitted for each fiber’s C 1s spectrum; however, significant variation was observed (Figure S4). TS30 fibers were predominately composed of C=C bonds (284.8 eV) and no C–C peak/C–H (285.1 eV) was present. Carbon–oxygen bonds contributed much less to the C 1s peak than did carbon–carbon bonds, suggesting that the oxide groups present on the TS30 may be more complex, such as phenols and quinones. C–O bonds contributed the majority of carbon–oxygen functionalization (286.5 eV), with minimal C=O presence (287.8 eV). However, COOH groups (289.0 eV) were present in small quantities but were absent from MS40 and HS40 fibers. MS40 and HS40 fibers had decreased C=C bonding, with C–C/C–H bonds composing the majority of the C 1s peak. Both fibers also showed a significant increase in carbon–oxygen bond contributions; C–O content doubled and C=O groups increased. While no COOH peak was observed on either MS40 or HS40 fibers, the C=O peak on the HS40 was particularly increased. Similar trends were observed on the O 1s peak (Figure S5). C–O bonds were the primary contributor to oxide functionalization on the TS30 and MS40 fibers; however, a shift towards C–OH groups was observed on HS40 fibers. Taken together, this suggests that contrary to conventional wisdom, these more graphitic fibers may have more oxide groups available for specific adsorption. In particular, HS40 fibers may facilitate enhanced adsorption at hydroxyl groups.

### E2 Detection is Nuanced at Carbon Fibers

Having characterized the surface structure and functionalization of our electrode fibers, we will now approach the electrode-analyte interface from the perspective of our target molecule. Previous characterization of E2 on TS30 fibers with FSCV yields a complex set of oxidations that present several problems for sensitive and prolonged monitoring in tissue matrices.^14,18^ E2 undergoes a primary (1°) oxidation that appears at 0.92 V when scanning at 400 V/s; the peak is shifted significantly past its oxidation potential due to scan rate outrunning sluggish electron transfer kinetics (Figure 2A,B). Its secondary (2°) oxidation appears at 1.3 V on the reverse scan, likewise pushed back by the fast scan rate. The primary peak’s reaction mechanism requires two 1-electron transfer steps sandwiching a ring rearrangement to facilitate the second electron loss from a phenoxy radical to a ketone (Figure 2C). This reaction is slow enough that it is visible at fast scan rates, as seen by the shoulder formed on the primary oxidation peak (Figure 2B; primary oxidation is divided into steps 1a and 1b). However, the hydroxy radical is highly unstable and thus, significant analyte loss is incurred between the electron transfer steps as the radicals form a dimer that can foul the electrode surface, decreasing surface area and available adsorption sites (Figure 2A,B, denoted as “Fouling”). Similar fouling peaks appear for other molecules prone to electropolymerization, such as serotonin and tryptophan.^30,40,41^ While significant negative current is often apparent at low potentials on the forward scan, previous work has established that this is nonFaradaic.^18^ Our electrochemical analysis will focus on facilitating E2 adsorption and increased surface coverage, and will also investigate electron transfer kinetics, sensitivity, and limit of detection.

**Figure 2:**
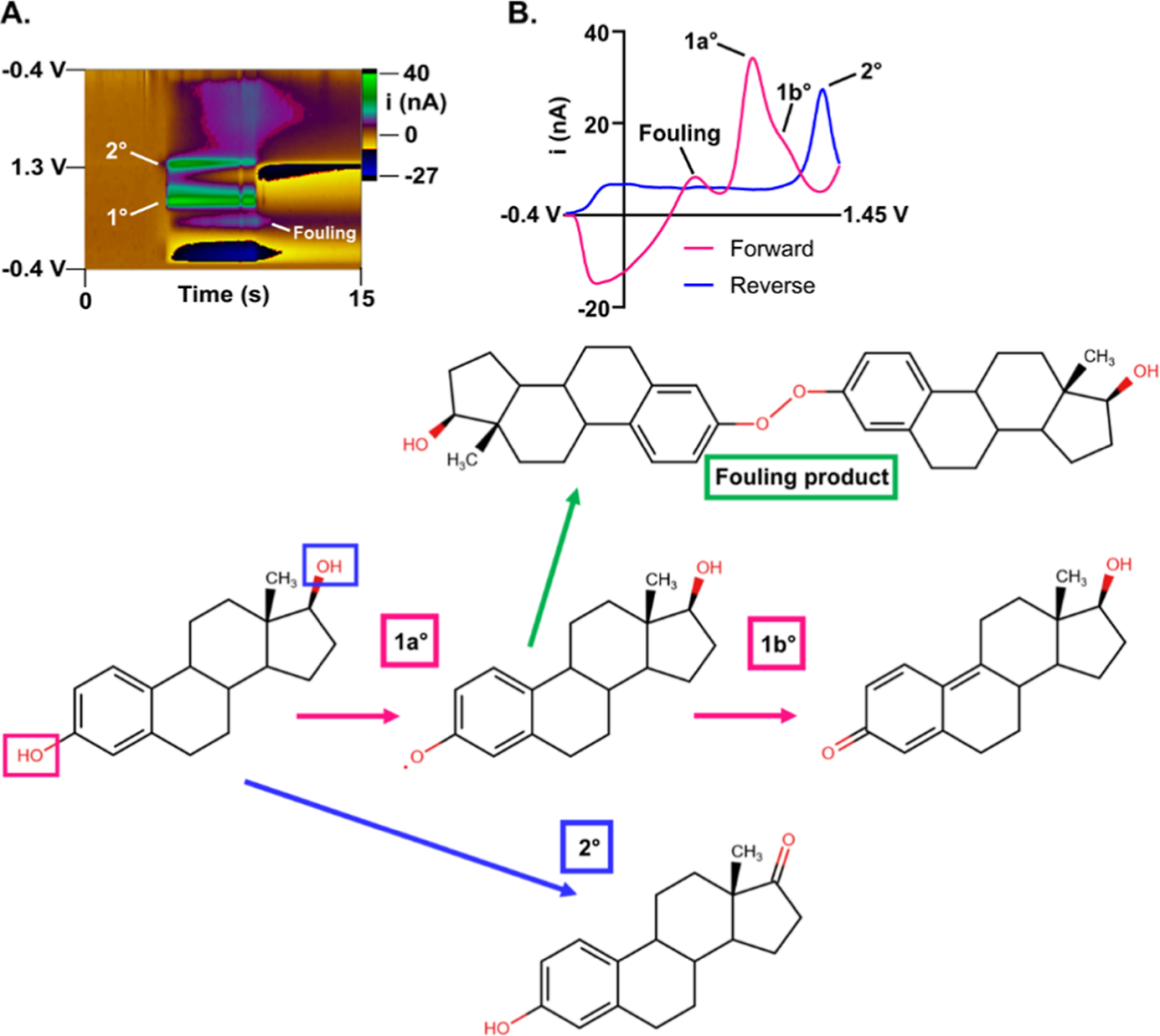
Estradiol undergoes a complex set of oxidations when measured using FSCV. (A) A representative false color plot shows a primary and secondary oxidation as well as a nonFaradaic fouling peak on a TS30 fiber. (B) E2’s cyclic voltammogram shows sluggish electron transfer on the primary oxidation peak. (C) E2’s proposed oxidation mechanism includes an ECE 2-electron transfer primary oxidation, a one-step 2-electron transfer secondary oxidation, and the formation of a dimer biproduct from the phenoxy radical intermediate generated during the first step of the primary oxidation.

### Characterization of E2 Adsorption

E2 is known to strongly adsorb at diverse carbon surfaces, and we elucidate its mass transfer limitation at TS30, MS40, and HS40 fibers by varying scan rate and waveform application frequency. Scan rates were varied from 50 V/s to 800 V/s and were plotted using a log–log plot (Figure 3A, B; *n*_TS30_ = 7, *n*_MS40_ = 5, *n*_HS40_ = 7). While conventional electrochemical techniques utilize Randles-Ševčik plots, high scan rates can overwhelm other contributing factors and prevent meaningful distinction between scan rate and the square root of scan rate (Figure S7). Slope of log–log plots was used to determine mass transfer limitation: complete adsorption limitation has an ideal slope of 1.0, while complete diffusion limitation has a slope of 0.5. For both the primary and secondary oxidations, TS30 fibers showed moderate adsorption limitation (*m*_1°‑TS30_ = 0.797 ± 0.032, *R*_1°‑TS30_^2^ = 0.993; *m*_2°‑TS30_ = 0.822 ± 0.012, *R*_2°‑TS30_^2^ = 0.999). For both reactions, MS40 and HS40 fibers moved towards a diffusion-limited regime. At MS40 fibers, both the primary and secondary oxidations’ slopes decrease to ∼0.6 (*m*_1°‑MS40_ = 0.631 ± 0.034, *R*_1°‑MS40_^2^ = 0.988; *m*_2°‑MS40_ = 0.623 ± 0.006, *R*_2°‑MS40_^2^ = 0.999). However, at HS40 fibers the reactions demonstrate opposing behavior: the primary peak shifts back towards adsorption limitation, while the secondary peak shows almost complete diffusion limitation (*m*_1°‑HS40_ = 0.701 ± 0.057, *R*_1°‑HS40_^2^ = 0.974; *m*_2°‑H40_ = 0.569 ± 0.046, *R*_2°‑HS40_^2^ = 0.974). This provides the first indication that E2’s phenol and hydroxyl moieties utilize individual binding sites rather than competing to adsorb.

**Figure 3:**
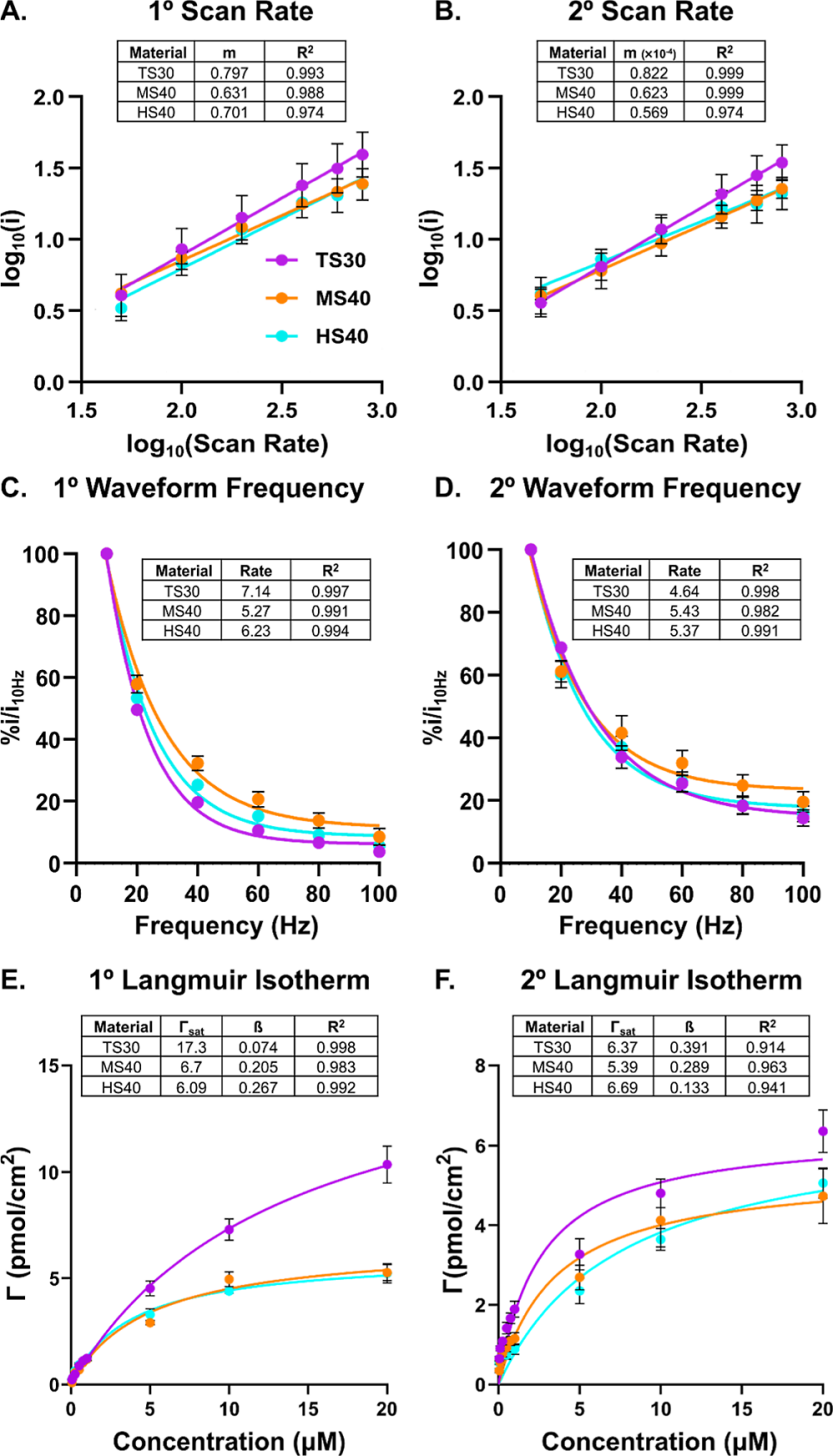
Electrochemical analysis with FSCV demonstrates that E2 adsorbs preferentially at more amorphous carbon at high speeds. Scan rate analysis shows strong adsorption limitation for both peaks at TS30 fibers that decreases with higher Young’s modulus (A,B). Increasing waveform application frequency decreases E2 adsorption and implies variation in adsorption speed across different fibers (C,D). Surface coverage is maximized at TS30 fibers for both the primary and secondary oxidations (E,F). (scan rate: *n*_TS30_ = 7, *n*_MS40_ = 5, *n*_HS40_ = 7, frequency: *n*_TS30_ = 5, *n*_MS40_ = 6, *n*_HS40_ = 7, *n*_langmuir_ = 7 TS30, *n*_langmuir_ = 6 MS40, HS40).

Varying the waveform application frequency further supports this supposition (Figure 3C, D; *n*_TS30_ = 5, *n*_MS40_ = 6, *n*_HS40_ = 7). As the waveform application frequency increases, the preaccumulation period decreases; at 100 Hz, each waveform is separated by only 0.75 ms. Completely diffusion limited analytes should experience minimal changes in current, while adsorption limited species show a sharp drop in current with increased frequency. Both the primary and secondary oxidations show significantly decreased current with increased frequency; doubling the frequency from 10 to 20 Hz decreased current by approximately 50% across all materials. This suggests that across all fibers, E2 operates in a strictly adsorption limited regime, contrary to the apparent diffusion shift seen for the MS40 and HS40 fibers from the scan rate experiment. We rationalize this as reflective of FSCV’s millisecond-temporal resolution paradigm. Assuming adsorption is our rate limiting step, slow adsorption processes may require several milliseconds to achieve significant surface coverage. Consequently, increasing the preconcentration period—as achieved with faster scan rates—allows slower processes to occur fully, while decreasing preconcentration may prohibit them entirely. Therefore, we propose that the apparently diffusion-limited behavior observed on the MS40 and HS40 fibers may reflect faster adsorption speeds, potentially due to increased efficacy of π–π stacking on more conjugated surfaces. We fit normalized current with an exponential one-phase decay model and calculated the rate of current loss; higher decay rates indicate sharper decreases in current with shorter adsorption times, indicating slower adsorption processes. For the primary oxidation, TS30 fibers showed the fastest rate of decay, while MS40 showed the slowest (REML mixed models with Bonferroni post hoc, *p*_TS30vsMS40_ < 0.0001, *p*_TS30vsHS40_ = 0.0012, *p*_MS40vsHS40_ = 0.0013). We infer in addition to π–π stacking, the increased C–O and C–OH functionalization observed on MS40 and HS40 fibers may contribute to faster adsorption. In contrast, current decay was slowest on TS30 fibers for the secondary oxidation; however, the differences were small enough that no significant changes were observed (REML mixed models with Bonferroni post hoc, *p*_TS30vsMS40_ = 0.8009, *p*_TS30vsHS40_ > 0.9999, *p*_MS40vsHS40_ = 0.1507). The divergent behavior observed between the two oxidations indicates that each oxidizable moiety likely adsorbs independently, rather than both the phenol and hydroxyl adsorbing concomitantly.

E2 adsorption was not enhanced by increased oxide surface functionalization. We utilized an electrochemical pretreatment waveform to increase hydroxyl and carbonyl moieties^37^ on TS30 fibers and tested 5 μM E2 before and after treatment. No significant change in current was observed (Figure S9). We hypothesize that more complex oxide functionalities—phenols, epoxies, and quinones—may be estradiol’s preferred binding sites, rather than the more abundant hydroxyl and carbonyl groups activated here. These groups may enable more facile adsorption through π–π stacking. This may further mark a difference between how to approach E2 as an analyte compared to more traditional neurochemicals. For example, catecholamines adsorb easily to small oxide moieties present in high abundance at the edge plane, which enhances their sensitivity at more disordered surfaces than E2 prefers.

Surface coverage for both the primary and secondary peaks was determined using a Langmuir adsorption isotherm (Figure 3E, F). The 1° phenol and 2° hydroxyl moieties were assumed to adsorb selectively at independent binding sites. The electrochemically active surface area was determined from application of the Randles–Ševčik equation to a scan rate experiment with 5 mM RuHex on each fiber type (Figure S6). Surface coverage was determined using eqs 1 and 2 (Figure S8). TS30 fibers achieve substantially higher surface coverage for the primary oxidation than either high Young’s modulus fiber, reaching saturation at 17.28 pM/cm^2^, more than double the coverage achieved at the MS40 (6.70 pM/cm^2^) or HS40 (6.09 pM/cm^2^) (REML with Bonferroni post hoc, *p*_TS30vsMS40_ = <0.0001, *p*_TS30vsHS40_ = 0.0001, *p*_MS40vsHS40_ > 0.9999, *n* = 7 TS30: MS40, HS40 *n* = 6). Similarly, the thermodynamic equilibrium constant (β), which describes desorption rate, is significantly lower at TS30 (0.074 × 10^–2^ cm^3^/pmol) than either of the more graphitic fibers (β_MS40_ = 0.205 × 10^–2^ cm^3^/pmol, βH_S40_ = 0.267 × 10^–2^ cm^3^/pmol), indicating increased desorption at more disordered surfaces. This implies that TS30 fibers may be more fouling resistant than MS40 or HS40 for E2, further suggesting that E2’s adsorption sites may differ radically from more traditional FSCV analytes.^30^ Concomitantly, adsorption strength *b* is likewise maximized on TS30 fibers for the primary oxidation, with MS40 fibers showing the lowest adsorption strength (Figure S10). MS40 and HS40 fibers were hypothesized to enhance surface coverage and adsorption strength through facilitating π–π interaction. TS30’s maximized surface coverage counterindicates this supposition, instead implying an essential role for the more complex oxide functional groups that decorate the TS30 surface.

In contrast, the secondary oxidation demonstrates equivalent surface coverage between TS30 and MS40 fibers, further confirming an independent adsorption mechanism. Surface coverage is much lower across all fibers for the 2° oxidation compared to the 1°, indication a paucity of specific adsorption sites. This supports the conclusions drawn from electrochemical treatment of the carbon surface revealing that the significantly more common C=O and C–OH surface moieties do not substantially enhance detection. The secondary oxidation achieved slight variation in surface coverage across all three fibers (REML with Bonferroni post hoc, *p*_TS30vsMS40_ = 0.1460, *p*_TS30vsHS40_ = 0.3252, *p*_MS40vsHS40_ > 0.9999, Γ_sat‑TS30_ = 6.37 pM/cm^2^, Γ_sat‑MS40_ = 5.38 pM/cm^2^, Γ_sat‑HS40_ = 6.68 pM/cm^2^, *n* = 7 TS30; *n* = 6 HS40, MS40). Comparatively little variation in adsorption strength *b* or thermodynamic equilibrium constant β is observed between the fibers, indicating universally low surface affinity (β_TS30_ = 0.391 × 10^–2^ cm^3^/pmol, β_MS40_ = 0.289 × 10^–2^ cm^3^/pmol, βH_S40_ = 0.133 × 10^–2^ cm^3^/pmol). This reflects previous computational conclusions at graphene surfaces, which found the primary hydroxyl had higher affinity for the carbon surface.^42^ Taken together, this suggests the secondary oxidation has vastly different optimization needs for enhanced adsorption than the primary oxidation and should not be prioritized when designing tailor-made carbon surfaces.

### Impact on Sensitivity and Electron Transfer Kinetics

Two practical concerns for fast E2 detection in tissue are high sensitivity at nanomolar concentrations and improved electron transfer kinetics to clearly resolve the primary peak. TS30 fibers have previously demonstrated low nanomolar limits of detection and mediocre sensitivity to E2 on an extended waveform.^18^ A concentration curve spanning 50 nM to 20 μM was used to determine the sensitivity, linear range, and LOD for E2 across the three fibers (Figure S11). TS30 fibers were more than twice as sensitive as MS40 and HS40 due to increased surface area. When normalized to electrochemically active surface area, MS40 and HS40 fibers showed near identical sensitivities and twice that of TS30 (*m*_TS30_ = 2.82 × 10^5^ nA·μM^–1^·cm^–2^, *m*_MS40_ = 4.55 × 10^5^ nA·μM^–1^·cm^–2^, *m*_HS40_ = 5.18 × 10^5^ nA·μM^–1^·cm^–2^, *n*_TS30,_ *n*_MS40_, *n*_HS40_ = 6). For the primary oxidation, both TS30 and MS40 fibers had an extended working range between 50 nM–10 μM; conversely, HS40 electrodes’ linearity was limited to 50 nM–5 μM. This is likely attributable to their small surface area and therefore fewer—and thus more quickly saturated—adsorption sites. The primary oxidation had a lower limit of detection than the secondary oxidation across all fibers; however, between fiber types no significant differences were observed (one-way two-tailed ANOVA with Bonferroni post hoc; *p*_1°_ = 0.1062, *p*_2°_ = 0.9267, primary oxidation LOD: TS30 = 61.6 ± 17.6 nM, MS40 = 110.8 ± 19.8 nM, HS40 = 98.18 ± 9.872 nM; secondary oxidation LOD: TS30 129.9 ± 34.9 nM, MS40 = 147.4 ± 37.3 nM, HS40 = 135.4 ± 22.44 nM; *n*_TS30_ = 6, *n*_MS40_ = 6, *n*_HS40_ = 6).

As E2’s primary oxidation is particularly sluggish, an ideal electrode material would substantially improve its electron transfer kinetics for a more clearly resolved peak. Changes in peak potential were examined across scan rates from 50 to 800 V/s. The primary peak on the TS30 and MS40 fibers was shifted approximately 0.2 V further on the waveform than on the HS40 (Figure S8A, REML with Bonferroni post hoc, *p*_TS30vsMS40_ = 0.9508, *p*_TS30vsHS40_ = 0.0004, *p*_MS40vsHS40_ < 0.0001, *n*_TS30_ = 7, *n*_MS40_ = 5, *n*_HS40_ = 7). The secondary peak was much more weakly affected, but did experience a modest 0.05 V improvement at the HS40 over the TS30 (Figure S8B, REML with Bonferroni post hoc, *p*_TS30vsMS40_ = 0.1665, *p*_TS30vsHS40_ = 0.0396, *p*_MS40vsHS40_ = 0.3590). Graphitic carbons are frequently used as semiconductors and in catalysis due to their excellent electron transfer properties; it is unsurprising that the highly structured HS40 fibers demonstrate these characteristics as well. We suggest that clear, sharp primary peak resolution may best be achieved on graphene-based fibers for future optimization.

### Structural Contributions to E2 Detection

Having examined E2’s interactions with varying carbon surfaces, we now shift perspective to consider the impact of estradiol’s structure on its adsorption and detection at carbon fibers. As previously noted, E2’s oxidizable groups are sterically hindered from coadsorption at the electrode surface. However, we have elucidated that the primary and secondary hydroxyls adsorb non-competitively at independent binding sites. This raises the question: what drives E2’s association at the electrode surface from the perspective of the analyte?

To answer this, we conducted a structural analysis of estradiol and four sterol analogues: estrone (E1), estriol (E3), progesterone (P4), and corticosterone (CORT) (Figure 4A–E). Estrone replaces E2’s secondary hydroxyl with a ketone, eliminating the secondary oxidation. E3 contains two potential hydroxyls for the secondary oxidation. P4 is not oxidizable within our potential window and CORT is sterically hindered from any potential oxidation. We compare the current at the primary and secondary E2 oxidation potentials across all analogues. As expected, E1 does not have a secondary oxidation; the average current for its primary oxidation is 76.8 ± 6.9% of E2’s. Conversely, E3’s secondary oxidation current is amplified, and the primary oxidation current increases to 134.2 ± 7.0% of estradiol’s (Figure 4F–H; repeated measures two-tailed ANOVA with Bonferroni post hoc, *p*_E1vsE2_ = 0.0135, *p*_E3vsE2_ = 0.0178, *n* = 4). This indicates that despite the 1° phenol and 2° hydroxyl adsorbing independently, the 2° hydroxyl significantly contributes to the driving force behind 1° adsorption and increases surface coverage. We hypothesize that this is caused by local dipoles from each hydroxyl engaging in hydrogen bonding at the electrode surface and providing motive force across an electrostatic gradient. As expected, neither P4 nor CORT show Faradaic current. There is also no evidence of nonspecific adsorption, further supporting the hypothesis that hydroxyls provide the driving force.

**Figure 4:**
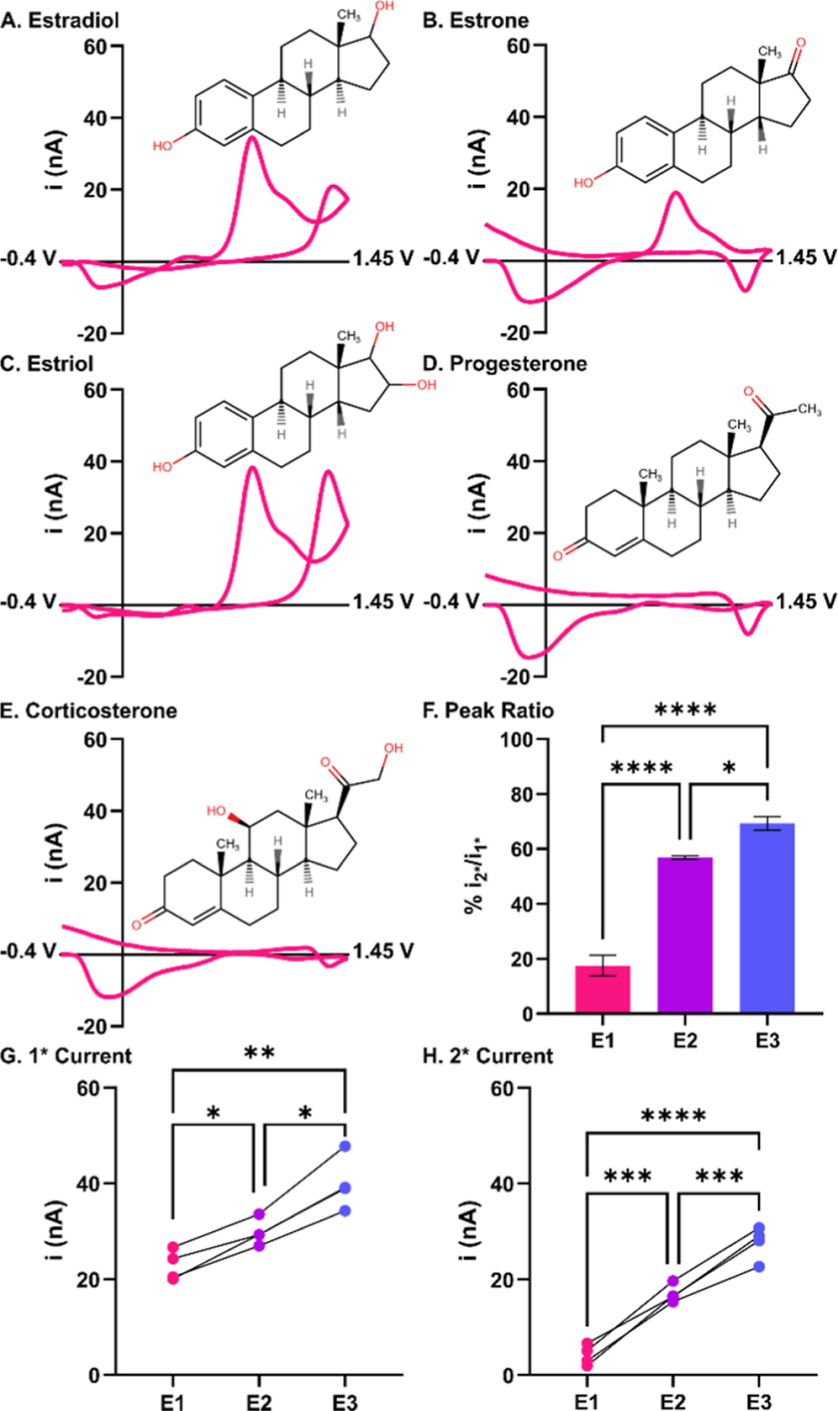
Estradiol’s hydroxyl moieties drive surface–analyte interactions and increase sensitivity. Comparison of 5 μM estradiol (A), estrone (B), and estriol (C) demonstrate a clear relationship between hydroxyl count and sensitivity. Progesterone (D) and corticosterone (E) are not oxidizable within the working potential window and did not show evidence of nonspecific adsorption. Current ratio between the primary and secondary peaks was increased for E3 oxidation compared to E2 (F). Both the primary (G) and secondary (H) peaks’ current increased with more hydroxyl functional groups on the analyte. *n* = 4.

From these findings, we see clear evidence that the entire molecular structure of estradiol is key to driving adsorption, not just its specific adsorption sites. Similar results have previously been observed with purines, where minor structural changes can significantly impact adsorption strength.^43^ However, no selectivity can be established for estradiol over estrone or estriol, which are also biologically relevant estrogens, as they undergo the same oxidation mechanism(s). Optimizing for selectivity is a delicate task for direct detection methods, and FSCV has historically established selectivity for target analytes through waveform modification as a result;^14,40,44,45^ however, this is often insufficient for similarly-structured analytes, which frequently adsorb to the same functional groups. While we do not establish selectivity over other similarly-structured analytes here, we demonstrate the crucial role surface structure plays in enhancing target analyte adsorption. On a broader scale, this highlights the necessity of tailoring electrode materials on an individual analyte level, as broad assumptions about classes of molecules may be insufficient for optimal detection.^32^ Here, we have streamlined an accessible approach to thorough optimization of the electrode-analyte interface for neurochemicals that present unique detection challenges (Figure S12); however, this approach may not be universally applicable to all neurochemicals with similar challenges. We also note a few limitations in our approach. While this work has discussed the cylindrical CFMEs used as homogeneous, electrochemistry and surface functionalization can often differ between the electrode tip and side walls, and FSCV is rapidly expanding to more novel electrode geometries than simple cylinders. Further work is necessary to understand the role of electrode geometry on E2 detection. Additionally, this work has not been validated in a live tissue matrix, as we suggest further improvement to electrode materials are necessary for sensitive and selective real time monitoring of E2. Previous work from our lab has validated E2 detection in tissue at a modified waveform, and future materials should include biological validation prior to employment studying in/ex vivo neurochemical release.

## Conclusion

Estradiol is a molecule that resists detection at conventional carbon surfaces at fast scan rates. Its mediocre adsorption and sluggish electron transfer hinder detection and its charge-neutral status prevents strong electrostatic effects from driving surface interaction. Here, we investigate the effects of specific structural differences on E2 detection and describe a roadmap for optimizing the electrode-analyte interface. Our results suggest that contrary to conventional wisdom, E2 adsorption is not dependent solely on π–π interactions and is likely driven by association at complex oxide binding sites, rendering customary carbon fiber pretreatments ineffective. Instead, we propose that E2 requires an electrode surface tailored to its specific needs—one that maintains fast electron transfer and is rich in oxygen-based defect sites beyond the customary carbonyl and hydroxyl functionalization. Graphene oxide fibers present an attractive possibility for enhanced E2 detection. Previous work suggests that GO nanostructures can significantly improve E2 detection at less effective carbon surfaces; we suggest that optimal detection requires more than just surface deposition—it requires a uniformly enhanced surface. We assert that sensitive, prolonged monitoring of neurochemical dynamics—including and beyond estradiol—mandate a customized, interface-prioritizing approach to detection. By considering the properties of the carbon surface, the electrochemical interactions from the target analyte, and the impacts of the analyte’s structure, we create a detailed picture of the ideal carbon surface for tailored, optimized detection. This approach is generalizable and accessible, providing a point of easy entry for optimization with a wide array of neurochemicals. In particular, the use of carbon fibers with varied surface characteristics enables faster and more affordable optimization than carbon materials such as CNTs and graphene derivatives normally allow. Our approach is particularly valuable for optimization of charge-neutral and negatively-charged analytes, which frequently cannot be easily measured with FSCV even after waveform optimization. As the field of rapid neurochemical monitoring expands—including FSCV, single-cell amperometry, and other electrochemical techniques—analyte-tailored sensing is increasingly necessary for confident detection of elusive target molecules.

## Supplementary Material



## Notes

S1: D/G ratio comparison across TS30, MS40, and HS40 fibers (Raman) S2: TS30 D/G mapping by intensity, MS40/HS40 mapping by area under D/G curve (Raman) S3: XPS oxygen content for TS30, MS40, HS40 fibers S4: XPS C 1s spectra with peak fitting for TS30, MS40, HS40 fibers S5: XPS O 1s spectra with peak fitting for TS30, MS40, HS40 fibers S6: RuHex square root of scan rate vs current for electrochemically active surface area S7: Scan rate vs. current and square root of scan rate vs current S8: Peak potential as a function of scan rate S9: Pre/post electrochemical treatment S10: Linear range of Langmuir isotherms S11: Concentration curve and limit of detection S12: Flowchart of optimization process (PDF)
