# Supplementary material for: Optimization of Subsecond Estradiol Detection through Analysis of Surface–Analyte Interactions
Source: ACS Electrochem. 2026 Mar 28;2(5):1262–72. doi: 10.1021/acselectrochem.6c00007 (PMC13158911; doi:10.1021/acselectrochem.6c00007)
Supplement: Supplementary file 1 [file ec6c00007_si_001.pdf]

Supporting Information

Optimization of Subsecond Estradiol Detection through Analysis of Surface-Analyte Interactions

Moriah E. Weese-Myers[a]+, Vivek Subedi[a]+, Naimah El-Amin[a]+, Faith Idahosa[a], Manisha Fowler[a], Ashley E. Ross[a]*

[a]

Department of Chemistry

University of Cincinnati

312 College Dr.

404 Crosley Tower

Cincinnati, OH 45221-0172

Email: ross2ah@ucmail.uc.edu

* corresponding author

+ equal contributions

1. Fast scan cyclic voltammetry as an electroanalytical technique

2. Raman analysis and mapping

Figure S1: D/G ratio comparison

Figure S2: TS30 D/G mapping by intensity, MS40/HS40 mapping by area under D/G curve

3. XPS C1s and O1s spectra and peak analysis

Figure S3: Oxygen content

Figure S4: C1s spectra with peak fitting for TS30, MS40, HS40 fibers

Figure S5: O1s spectra with peak fitting for TS30, MS40, HS40 fibers

4. Electrochemically active surface area

Figure S6: RuHex square root of scan rate vs current for electrochemically active surface area

5. Mass transfer limitation and electron transfer kinetics

Figure S7: Scan rate vs. current and square root of scan rate vs current

Figure S8: Peak potential as a function of scan rate

6. Impact of increased oxide surface functionality

Figure S9: E2 pre/post electrochemical treatment

7. Langmuir isotherms

Equations 1-4: Langmuir isotherm calculation

Figure S10: Linear range of Langmuir isotherms

8. Electrode sensitivity to E2

Figure S11: Concentration curve and limit of detection

9. Optimizing electrode-analyte interactions

Figure S12: Flowchart of optimization process

1. Fast scan cyclic voltammetry as an electroanalytical technique

Fast scan cyclic voltammetry (FSCV) is an electroanalytical technique typically performed at carbon fiber microelectrodes that utilizes scan rates on the order of hundreds of volts per second for subsecond monitoring of low nanomolar analyte concentrations. As the name suggests, it utilizes a conventional potential sweep to oxidize and reduce target analytes. Waveform application time is frequently under 10 ms per sweep, resulting in a very thin diffuse layer. Consequently, FSCV differs from conventional CV by utilizing an extended preconcentration period which is up to 90 ms long, enabling extended analyte accumulation. Fast scan rates generate significant capacitive current, necessitating the use of ultramicroelectrodes. It also requires background subtraction for detection of nanoampere levels of current, resulting in a technique best suited for monitoring fast fluctuations but incapable of studying basal analyte levels.

2. Raman analysis and mapping

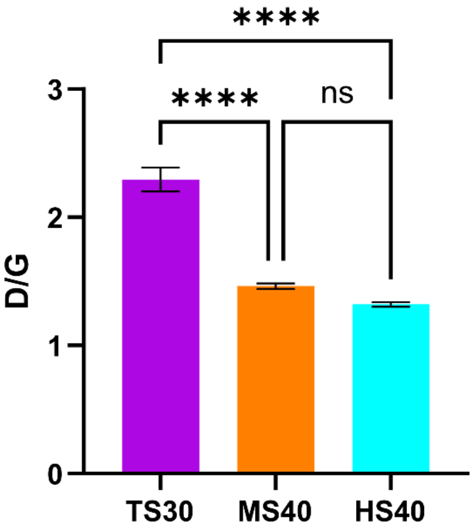

Figure S1. TS30 fibers are significantly more disordered that MS40 or HS40 fibers. The D/G ratio was averaged across 121 measurements per sample per fiber type (*n* = 6 all fibers). TS30 surfaces are highly disordered, while MS40 and HS40 surfaces show less structural disorder (one-way two-tailed ANOVA with Bonferroni post hoc, $p < 0.0001$ all comparisons).

Conventionally, the D/G ratio is determined using peak intensity. However, amorphous carbon has broad, convoluted D and G peaks that can artificially increase the G peak intensity. Work by Ferrari and Robertson concludes that disordered and amorphous carbons should be analyzed using the area of the D and G peaks, not the intensity[1,2]. In this work, we analyze TS30 fibers using peak area and MS40 and HS40 fibers using peak intensity (Figure 1D-F). Below are the alternative Raman maps for each fiber: peak intensity for TS30 and peak area for MS40 and HS40.

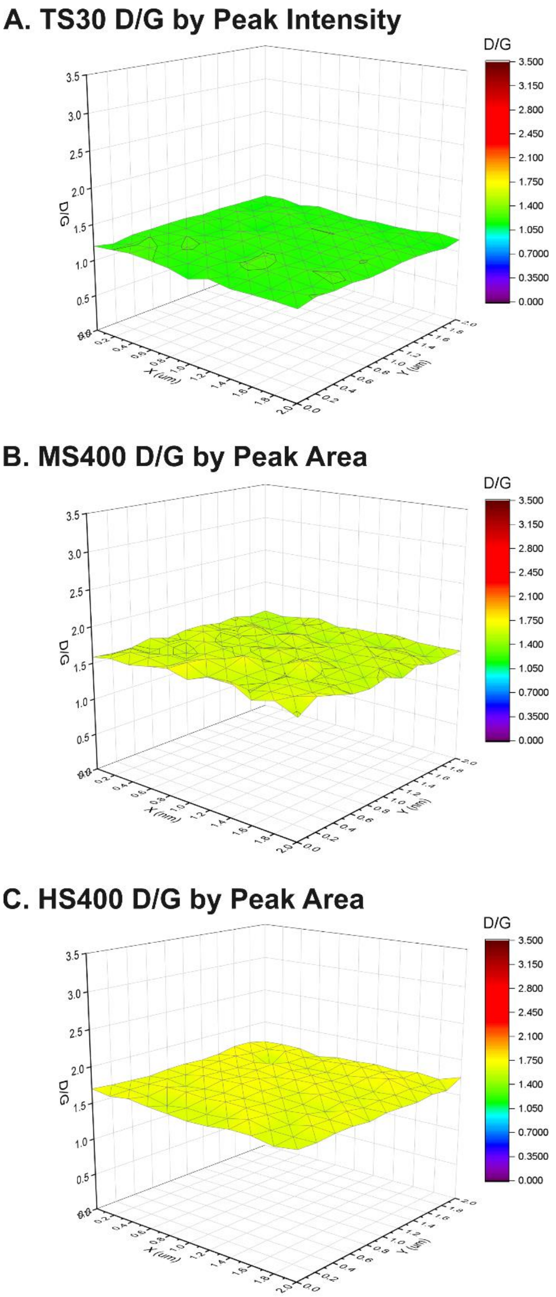

Figure S2. TS30 D/G mapping by intensity, MS40/HS40 mapping by area under D/G curve. The D/G ratio is artificially reduced for TS30 fibers due to peak convolution (A). Conversely, MS40 (B) and HS40 (C) D/G ratios are increased, overestimating the degree of disorder on the surface.

3. XPS C1s and O1s spectra and peak analysis

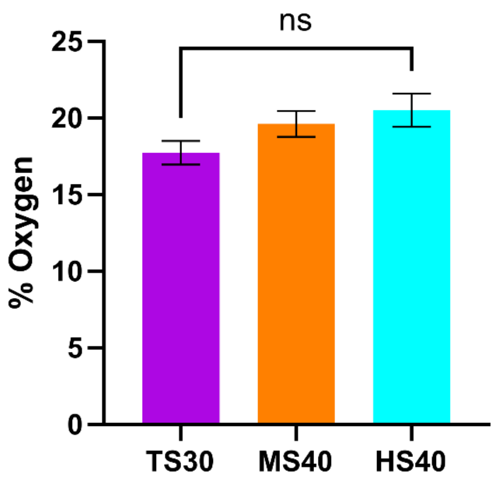

Figure S3. Total oxygen content does not change between TS30, MS40, and HS40 fibers (one-way two-tailed ANOVA with Bonferroni post hoc, p =.1154, n = 7 all fibers). Oxygen content was determined by integrating the C1s and O1s peaks for each fiber. Oxygen accounted for 17.7 ± 0.8 % of TS30 surface composition, 19.6 ± 0.8 % of MS40, and 20.5 ± 1.0 % of HS40.

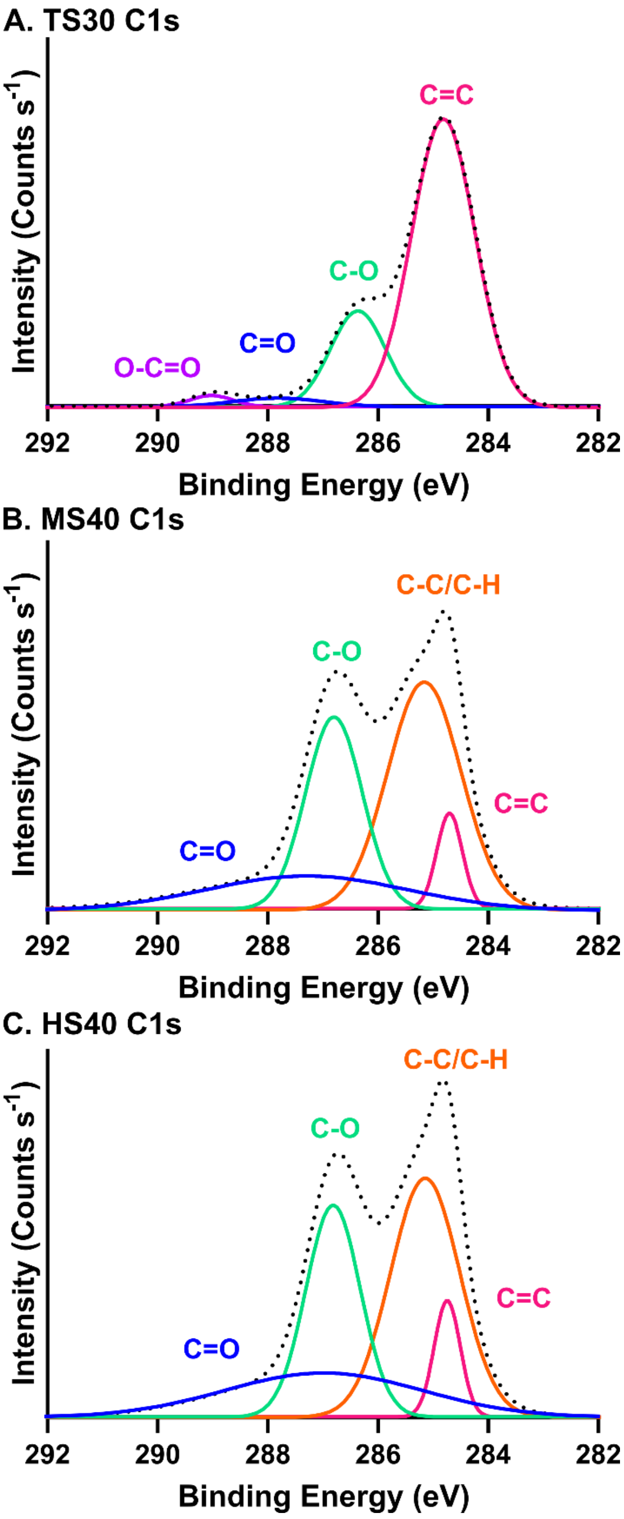

Figure S4. XPS C1s peak analysis shows that carbon composition and functionalization changes between TS30, MS40, and HS40 fibers. A) TS30 fibers have COOH surface functionalization. B) MS40 fibers show a significant shift towards C-C/C-H bonding and increased C-O and C=O functionalization. C) HS40 fibers show similar composition to MS40 but have proportionally fewer C-C/C-H bonds.

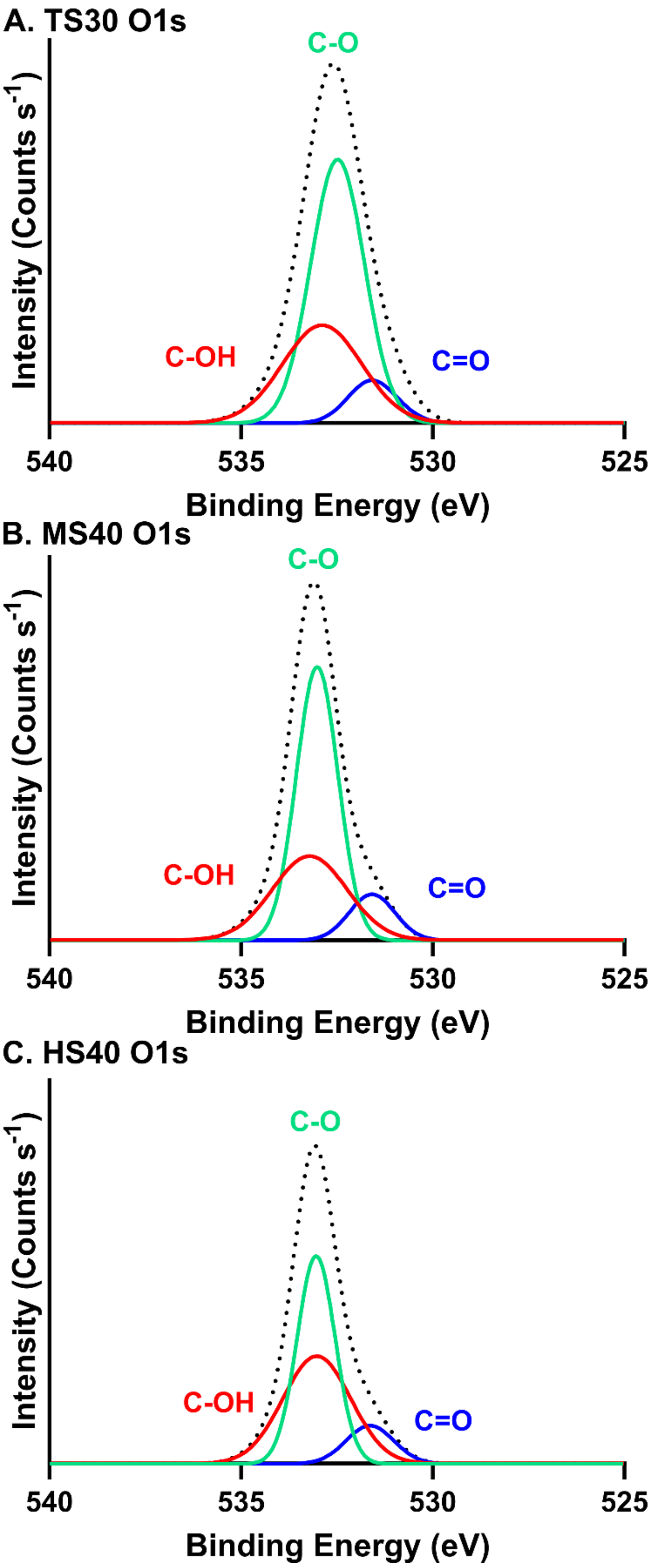

Figure S5. XPS analysis of the O1s peak reveals subtle changes in surface oxide functionalization between TS30 (A), MS40 (B), and HS40 (C) fibers. TS30 fibers have proportionally more C-O moieties than MS40 and HS40. MS40 fibers show small increases in C=O bonding while HS40 fibers instead show increased C-OH functionalization.

4. Electrochemically active surface area

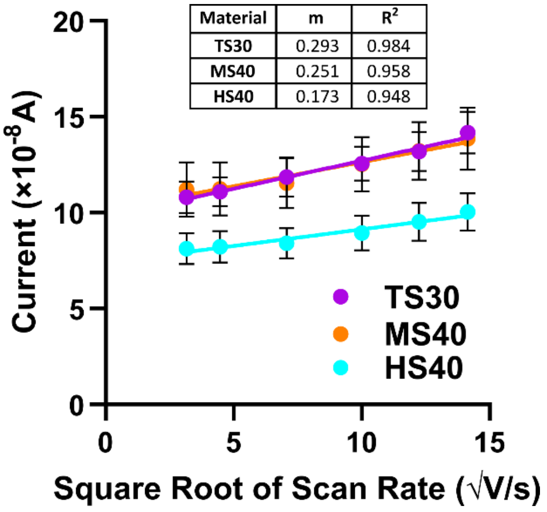

Figure S6. Electrochemically active surface area (ESCA) was calculated from the slope of the square root of scan rate for 5 mM RuHex on TS30, MS40, and HS40 electrodes using the Randles-Ševčik equation. On all fibers, the ESCA was lower than the geometric surface area (n = 3 all fibers).

5. Mass transfer limitation and electron transfer kinetics

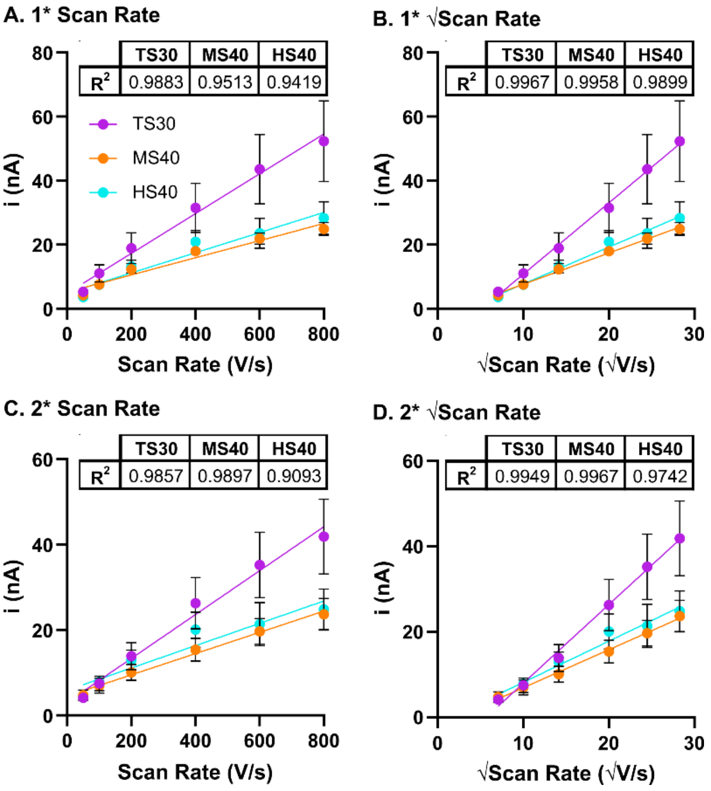

Figure S7. Randles-Ševčik plots for the primary and secondary oxidations. Mass transfer limitation is traditionally determined by the linearity of scan rate (A, C) and square root of scan rate (B, D) vs current. High linearity is observed across all fibers for both oxidations with both models, necessitating the use of log-log plots to clarify the mass transfer limiting step. $n_{TS30} = 7$, $n_{MS40} = 5$, $n_{HS40} = 7$

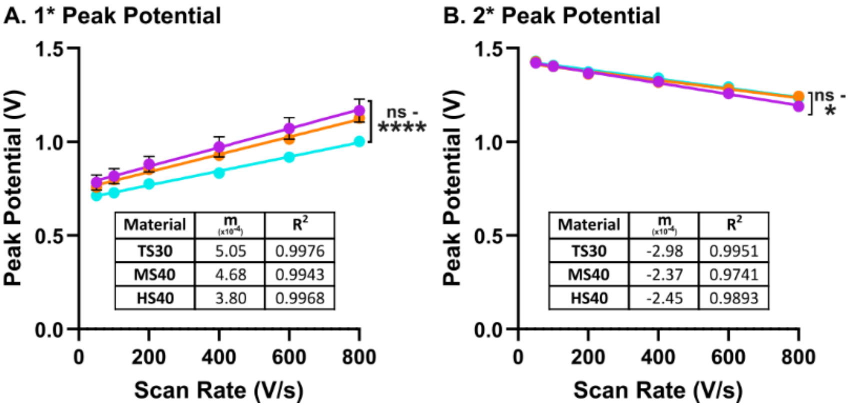

Figure S8. Peak potential for primary and secondary oxidations as a function of scan rate. A) HS40 fibers showed significantly faster electron transfer than TS30 and MS40 electrodes for the primary peak (REML with Bonferroni post hoc, $p_{TS30 vs MS40} = 0.9508$, $p_{TS30 vs HS40} = 0.0004$, $p_{MS40 vs HS40} < 0.0001$). B) Much smaller effects were seen at the secondary oxidation, with HS40 fibers slightly improving electron transfer over TS30 fibers (REML with Bonferroni post hoc, $p_{TS30 vs MS40} = 0.1665$, $p_{TS30 vs HS40} = 0.0396$, $p_{MS40 vs HS40} = 0.3590$). $n_{TS30} = 7$, $n_{MS40} = 5$, $n_{HS40} = 7$

6. Impact of increased oxide surface functionality

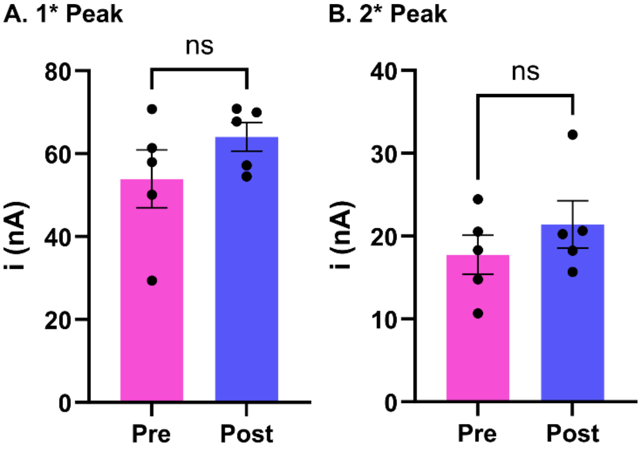

Figure S9. Electrochemical treatment does not increase E2 sensitivity. A waveform scanning from -0.5 V to 1.8 V at 400 V/s and 10 Hz was applied to TS30 electrodes for 5 minutes. No significant differences in average current were observed for the primary (A) or secondary (B) oxidations, although current trended upwards by ~20% following treatment for both oxidations (paired t-test, $p_{1°} = 0.0889$, $p_{2°} = 0.0935$, $n = 5$).

7. Langmuir isotherms

Surface coverage was calculated using the following equations:

(1) $\Gamma_A = \frac{\Gamma_{sat}\beta[E2]}{1+\beta[E2]}$

(2) $i_p = \frac{n'^2\theta F^2}{2.718RT} vA\Gamma$

Γ_A is surface coverage, Γ_{sat} is the saturation surface coverage, and β is the adsorption thermodynamic equilibrium constant. Both E2 oxidations are irreversible, so eq. 2 was used to calculate Γ_A. In eq. 2, i_p is peak oxidative current, n' is the number of electrons transferred before an irreversible step, θ is the total number of electrons transferred divided by n', F is Faraday's constant, v is scan rate, and A is electrode surface area. For the 1° oxidation, n' = 1 and θ = 2; for the 2° oxidation, n' = 2 and θ = 1. Electrode surface area was calculated for each fiber type based on average diameter (TS30 = 8.5 µm, MS40 = 6 µm, HS40 = 5 µm); all electrodes for this experiment were cut between 80-100 µm, averaged to 90 µm. Surface area for TS30 fibers was 2.46×10^{-4} cm^2, for MS40 was 1.73×10^{-5} cm^2, and for HS40 was 1.43×10^{-5} cm^2.

At low concentrations, Γ_A <<< Γ_{sat}, allowing use of a linearized form of eq. 1:

(3) $\Gamma_A = b[E2]$

where

(4) $b = \Gamma_{sat}\beta$

Eq. 3's slope b is an equilibrium coefficient that determines adsorption strength.

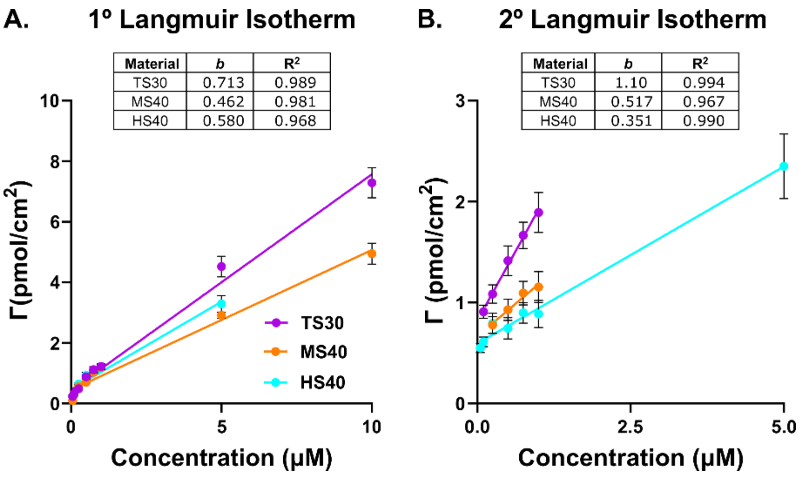

Figure S10. Linear range of surface coverage for primary (A) and secondary (B) peaks. Equilibrium coefficient b is defined as the slope of the linear range. For the primary oxidation, TS30 fibers were linear from 100 nM – 10 µM, MS40 from 100 nM – 10 µM, and HS40 from 100 nM – 5 µM. For the secondary

oxidation, TS30 were linear from 100 nM – 1 μM, MS40 from 250 nM – 1 μM, and HS40 from 50 nM – 5 μM. *n* = 6 TS30, MS40, HS40

8. Electrode sensitivity to E2

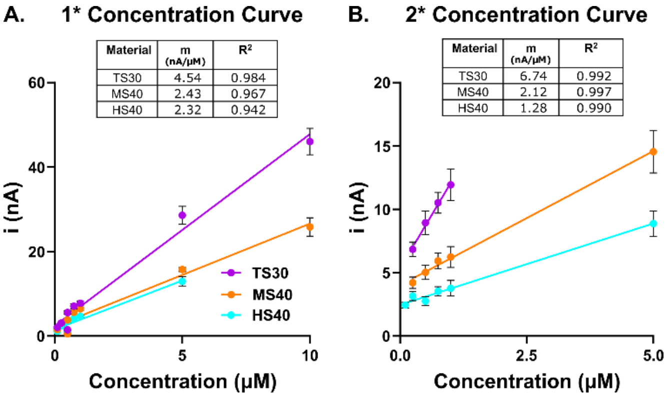

Figure S11. Sensitivity is maximized on TS30 fibers due to increased surface area for both the primary (A) and secondary (B) oxidations. For the primary oxidation, TS30 fibers were linear from 50 nM – 10 μM, MS40 from 50 nM – 10 μM, and HS40 from 50 nM – 5 μM. For the secondary oxidation, TS30 were linear from 250 nM – 1 μM, MS40 from 250 nM – 5 μM, and HS40 from 100 nM – 5 μM. (*n* = 6 TS30, MS40, HS40)

9. Optimizing electrode-analyte interactions

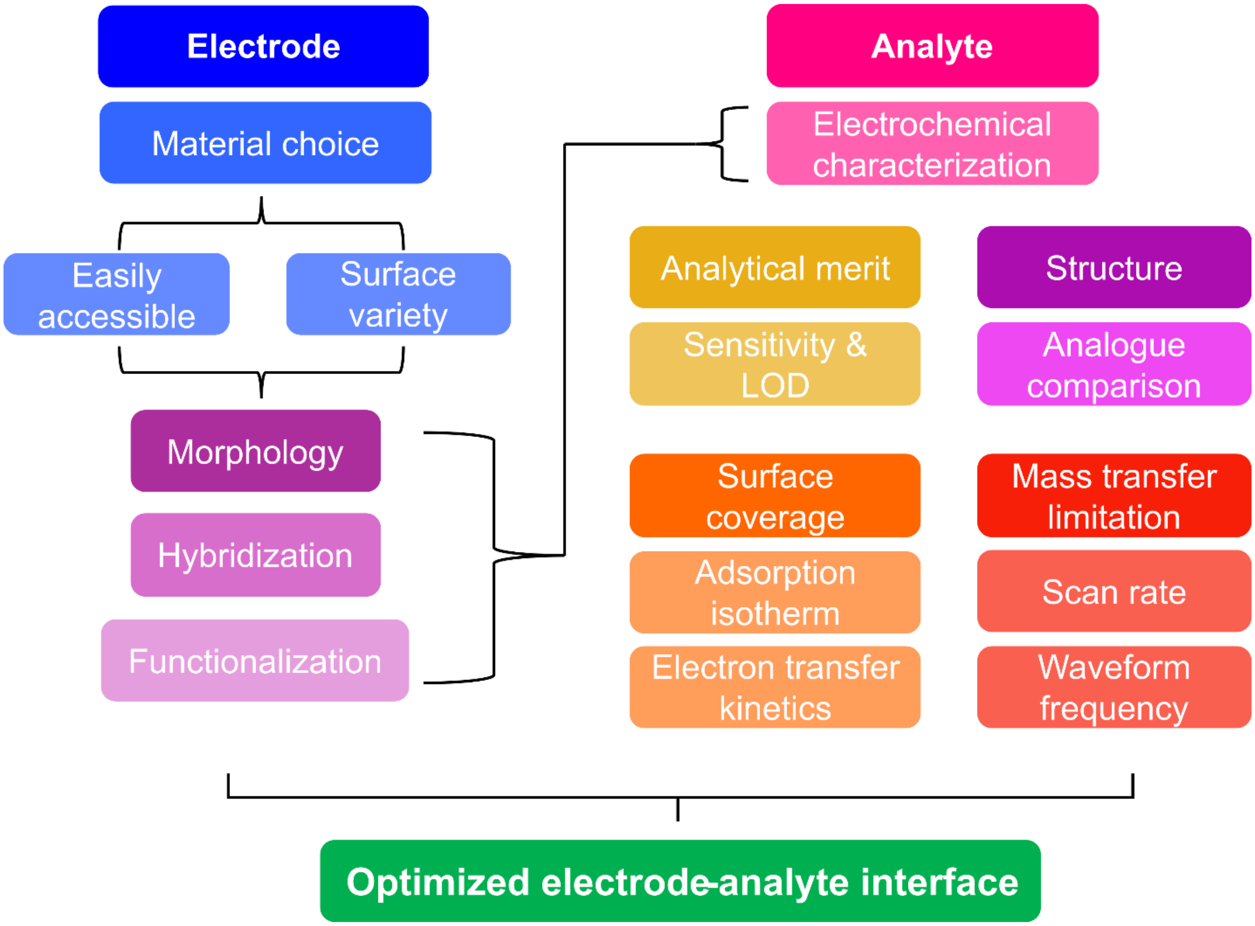

Figure S12. In this work we present a streamlined approach to optimizing the electrode-analyte interface for neurochemicals that prove challenging to direct detection. Our approach is outlined above as a two-pronged method that utilizes readily available materials to determine the ideal detection surface for specific analytes.

[1] A. C. Ferrari, J. Robertson, "Interpretation of Raman spectra of disordered and amorphous carbon" *Phys. Rev. B* **2000**, *61*, 14095–14107.

[2] A. C. Ferrari, J. Robertson, "Resonant Raman spectroscopy of disordered, amorphous, and diamondlike carbon" *Phys. Rev. B* **2001**, *64*, 075414.
